# Short-term creatine supplementation changes protein metabolism signaling in hindlimb suspension

**DOI:** 10.1590/1414-431X20198391

**Published:** 2019-10-07

**Authors:** G.N. Marzuca-Nassr, M.A.S. Fortes, L. Guimarães-Ferreira, G.M. Murata, K.F. Vitzel, D.A.A. Vasconcelos, R.A. Bassit, R. Curi

**Affiliations:** 1 Department of Internal Medicine, Faculty of Medicine, Universidad de La Frontera, Temuco, Chile; 2 Departamento de Fisiologia e Biofísica, Instituto de Ciências Biomédicas, Universidade de São Paulo, São Paulo, SP, Brasil; 3 Grupo de Estudos em Fisiologia Muscular e Performance Humana, Centro de Educação Física e Desportos, Universidade Federal do Espírito Santo, Vitória, ES, Brasil; 4 School of Health Sciences, College of Health, Massey University, Auckland, New Zealand; 5 Departamento da Biologia Celular e do Desenvolvimento, Instituto de Ciências Biomédicas, Universidade de São Paulo, São Paulo, SP, Brasil; 6 Programa de Pós-graduação Interdisciplinar em Ciências da Saúde, Universidade Cruzeiro do Sul, São Paulo, SP, Brasil

**Keywords:** Creatine supplementation, Hindlimb suspension, Muscle disuse atrophy, Protein synthesis, Protein degradation

## Abstract

The effect of a short-term creatine supplementation on hindlimb suspension (HS)-induced muscle atrophy was investigated. Creatine monohydrate (5 g/kg b.w. per day) or placebo, divided in 2 daily doses, was given by oral gavage for 5 days. Rats were maintained in HS with dietary supplementation concomitantly for 5 days. Body weight, soleus and EDL muscle masses, and cross-sectional areas (CSA) of the muscle fibers were measured. Signaling pathways associated with skeletal muscle mass regulation (*FST*, *MSTN*, *FAK, IGF-1, MGF*, *Akt*, *mTOR*, *atrogin-1*, and *MuRF1* expressions, and Akt, S6, GSK3B, and 4EBP1 proteins) were evaluated in the muscles. Soleus muscle exhibited more atrophy than the EDL muscle due to HS. Creatine supplementation attenuated the decrease of wet weight and increased p-4EBP1 protein in the EDL muscle of HS rats. Also, creatine increased *mTOR* and *atrogin-1* expressions in the same muscle and condition. In the absence of HS, creatine supplementation increased *FAK* and decreased *MGF* expressions in the EDL muscle. Creatine attenuated the increase in *FST* expression due to HS in the soleus muscle. *MuRF1* expression increased in the soleus muscle due to creatine supplementation in HS animals whereas *atrogin-1* expression increased still further in this group compared with untreated HS rats. In conclusion, short-term creatine supplementation changed protein metabolism signaling in soleus and EDL muscles. However, creatine supplementation only slightly attenuated the mass loss of both muscles and did not prevent the CSA reduction and muscle strength decrease induced by HS for 5 days.

## Introduction

Skeletal muscle disuse atrophy is characterized by a decrease in activity of protein synthesis and/or an increase in protein degradation pathways leading to a reduction of muscle mass and strength and in the cross-sectional area (CSA) of muscle fibers (1,2). Several strategies have been tested to attenuate muscle wasting such as electrical stimulation (3), physical exercise (4), and dietary supplementation; e.g., fish oil, leucine, isoleucine, valine, creatine, and L-carnitine (2,5,6). Creatine may act as an attenuating agent of skeletal muscle atrophy (6) and leads to an increase of physical exercise capacity (7). However, a consensus about the beneficial effects of creatine dietary supplementation in skeletal muscle atrophy is still lacking.

Creatine is a non-essential dietary nutrient and is present in meat and fish. Endogenous creatine synthesis occurs in the liver, kidney, and pancreas after a two-stage process using arginine and glycine as precursors. Skeletal muscle is the main site for creatine storage where creatine kinase converts it into phosphocreatine with the use of ATP (8–10). Creatine supplementation has been reported to act on skeletal muscle atrophy through an increase in strength, resistance to fatigue, intramuscular phosphocreatine content, and protein synthesis markers. Creatine supplementation decreases cytoplasmic Ca^2+^ levels, production of reactive oxygen species, contents of proinflammatory cytokines, satellite cell activation, and muscle cell apoptosis (11–13).

Creatine supplementation (250 mg/kg b.w. per day for 18 days) attenuated muscle mass loss in corticosteroid-induced muscle wasting in rats (14), and increased fat-free mass and strength in myopathies such as McArdle's disease, mitochondrial chronic progressive external ophthalmoplegia (CPEO), mitochondrial encephalopathy, lactic acidosis, and stroke-like episodes (MELAS) in rodents (12,15). In mice, creatine supplementation (administered both in powder food (4.7%) and drinking water (1.4%) for 45 days) did not attenuate muscle atrophy after myotoxic injury (16) induced by dexamethasone treatment (17) or in a transgenic model of amyotrophic lateral sclerosis (18). In a condition of muscle atrophy induced by immobilization, creatine supplementation (5 g/kg b.w. per day for 14 days) attenuated muscle mass loss in rats (19). Creatine supplementation attenuated muscle mass loss in the upper-arm of young men (20) but had no effect in the leg muscles of human subjects after immobilization (21,22). Backx et al. (21) reported that, in healthy young men (mean of 23 years old) after muscle disuse atrophy caused by immobilization, creatine supplementation did not attenuate skeletal muscle loss and the decreased CSA of the quadriceps. These reports indicate the anti-atrophic effect of creatine is still controversial.

With the high incidence of chronic diseases and the increase in the aged population, there is an increased number of patients on short-term bed rest. The average hospitalization duration for adults is 5–7 days leading to a ∼0.6% loss of leg muscle mass per day (23). Taking this fact into consideration, the focus of this work was to evaluate the effects of a short-term creatine supplementation on muscle disuse. Due to the difficulty of developing bed-rest studies in humans, animal models were used. One of the most widely used experimental models to mimic the skeletal muscle loss induced by bed rest in humans is the hindlimb suspension (HS) in rodents.

We investigated the short-term effects of creatine supplementation on skeletal muscle signaling pathways involved in protein metabolism in rats with HS-induced atrophy. We hypothesized that short-term creatine supplementation would attenuate the skeletal muscle mass loss and muscle strength reduction by preventing the changes in protein metabolism signaling induced by short-term disuse.

## Material and Methods

### Animals

Eight-week-old male Wistar rats were obtained from the Animal Facility of the Department of Physiology and Biophysics, Institute of Biomedical Sciences, University of São Paulo. The animals were maintained under standard conditions of 12-h light/dark cycle at 23±2°C and food and water *ad libitum* (daily consumption was recorded). All experimental procedures were performed in accordance with the Guide for the Care and Use of Laboratory Animals (Institute of Laboratory Animal Resources, National Academy of Sciences, USA). Ethics Committee of the Institute of Biomedical Sciences, University of São Paulo, approved the experimental protocol and procedures of this study (No. 65/20/03).

### Experimental study design

During the first three days of the experimental period, the animals were adapted to individual cages. Afterwards, the rats were divided into the following groups: control group (C, n=7); hindlimb suspension group (HS, n=8); creatine supplemented group (Cr, n=7); and creatine supplemented and hindlimb suspension group (Cr-HS, n=8). The animals received a daily oral supplementation of creatine monohydrate (Merck, Darmstadt, Germany) by gavage (5 g/kg b.w.), divided in 2 daily doses for 5 days. Eijnde et al. (24) reported that this dosage promotes an increase of 30 and 15% of free creatine and phosphocreatine, respectively, in the soleus muscle after 5 days of supplementation in rats. Similar responses were seen in humans with the dose commonly used, 20 g creatine monohydrate per day for 5 days (25,26). The same volume of water was given as placebo to the C and HS groups.

The HS is a well-established experimental model for induction of skeletal muscle atrophy as previously described (2,27). Animals were submitted to HS and dietary creatine supplementation concomitantly. Rats were then maintained in the HS and dietary supplementation with creatine or placebo for 5 days. After that, the animals were weighed and anesthetized using ketamine (90 mg/kg b.w.) and xylazine (10 mg/kg b.w.) by intraperitoneal administration. We removed the soleus and extensor digitorum longus (EDL) muscles of both limbs for histological and molecular analysis (western blotting and real-time polymerase chain reaction – RT-PCR) and euthanized the animals by exsanguination.

### Analysis of muscle strength and fatigue resistance

An *in vivo* electrical stimulation protocol was used for determination of muscle contractile activity. For twitch force analysis, the stimulus consisted of 500-μs pulse at 1 Hz with adjusted voltage to produce maximum force. Electrical stimulus frequency was increased to 100 Hz to determine the tetanic force. Ten 1-s successive tetanic contractions at 100 Hz allowed the determination of fatigue resistance, with 10 s of recovery between them, by measuring the decrease in force production during the experimental protocol used. Maximal twitch and tetanic forces were recorded using the AqDados software (version 4.16, Lynx Tecnologia Eletronica Ltda., Brazil). Muscle strength and fatigue resistance were analyzed using the AqAnalysis software (version 4.16, Lynx Tecnologia Eletronica Ltda.). We used a similar procedure in previous studies (28).

### Histological analysis

Serial sections were taken from the central portion of the soleus and EDL muscles according to Bodine and Baar (29). The slides were stained with hematoxylin and eosin (HE) for analysis of CSA of the soleus and EDL muscles fibers (150 fibers per muscle). Photographs were taken using an optical microscope (Nikon Eclipse E1000, Japan) attached to a digital camera (Nixon DXM 1200). The images were analyzed using the AxioVision program (version 4.8.1.0, Carl Zeiss Imaging Solutions, Germany). We used the same measurements in a previous study (2).

### Analysis of Akt, S6, GSK3B, and 4EBP1 by western blot

The primary antibodies used were: p-protein kinase B (Akt) at Ser 473 (9271), Akt (9272), p-S6 at Ser 240/244 (5364), S6 (2217), p-GSK3B (glycogen synthase kinase3-beta) at Ser 9 (9323), GSK3B (9315), p-4EBP1 at Thr 37/46 (2855) and 4EBP1 (9644) from Cell Signaling Technology (USA). We used a similar procedure in previous studies (2,27,30).

### Real-time polymerase chain reaction (RT-PCR)

Total RNA was extracted from skeletal muscles using RNeasy RNA isolation kit (Qiagen Inc, USA) according to the manufacturer's protocol and as used in our previous study (28). The following genes were evaluated: *FST (*follistatin); *MSTN* (myostatin); *FAK* (focal adhesion kinase); *IGF-1* (insulin-like growth factor); *MGF* (mechano growth factor); *Akt*; *mTOR* (mammalian target of rapamycin); *atrogin-1*, and *MuRF1*. The primers sequences used in the experiments are displayed in Supplementary Table S1.

### Statistical analysis

Statistical analysis was performed using the GraphPad Prism^®^ software (version 4.01; USA). Results are reported as means±SE and were analyzed by two-way analysis of variance (ANOVA) followed by the Bonferroni *post-hoc* test (for comparison between three or more groups). Outlier results were detected using the Grubbs' test of GraphPad Software (graphpad.com/quickcalcs/Grubbs1.cfm). The differences between groups were considered significant for P<0.05.

## Results

### Body weight, and soleus and EDL muscles masses

The HS group had lower body weight than the other groups after 5 days of experiment (Table 1). HS reduced wet (P<0.001) and dry (P<0.001) soleus muscle mass by 29–33% in the untreated (C) group. In the EDL muscle, the wet weight was significantly decreased by 16% (P<0.05) by HS, while the dry weight was decreased by 12% (P<0.05). Creatine supplementation had an effect on preventing muscle mass loss that was more notable in the EDL muscle, in the rats submitted to HS (Table 1).

**Table 1:** Body weight and muscle masses of soleus and extensor digitorum longus (EDL) muscles.

Measurements	Groups			
	C	HS	Cr	Cr-HS
Body weight (g)				
Day –3	201±2.3	201±4.6	200±4.6	207±3.3
Day 0	222±3.2	220±6.1	221±10.5	224±4.3
Day 5	251±4.0	216±3.3^b^	251±9.0	206±11^c^
Soleus muscle				
Wet weight (mg/mm tibia length)	3.2±0.02	2.1±0.08^c^	3.1±0.04	2.2±0.01^c^
% loss due to HS in wet weight		33%		26%
Dry weight (mg/mm tibia length)	0.84±0.05	0.59±0.04^c^	0.77±0.02	0.63±0.04^a^
% loss due to HS in dry weight		29%		18%
EDL muscle				
Wet weight (mg/mm tibia length)	3.8±0.08	3.2±0.01^a^	3.3±0.02	3.0±0.02
% loss due to HS in wet weight		16%		9%
Dry weight (mg/mm tibia length)*	0.97±0.02	0.85±0.03	0.86±0.04	0.8±0.04
% loss due to HS in dry weight		12%		8%

Data are reported as means±SE, n=7–8 animals. The results were compared using two-way ANOVA and Bonferroni *post hoc* test. ^a^P<0.05; ^b^P<0.01; ^c^P<0.001: significant differences between hindlimb suspension groups and the respective controls (C *vs* HS or Cr *vs* Cr-HS). *P<0.05 for C and Cr groups *vs* C-HS and Cr-HS groups (main effects of HS), using two-way ANOVA only (no statistical differences using the Bonferroni *post hoc* test). C: control group; HS: hindlimb suspension group; Cr: creatine supplemented group; Cr-HS: creatine supplemented and hindlimb suspension group; EDL: extensor digitorum longus.

### Analysis of strength and fatigue resistance in the soleus and EDL muscles

**Figure 1:**
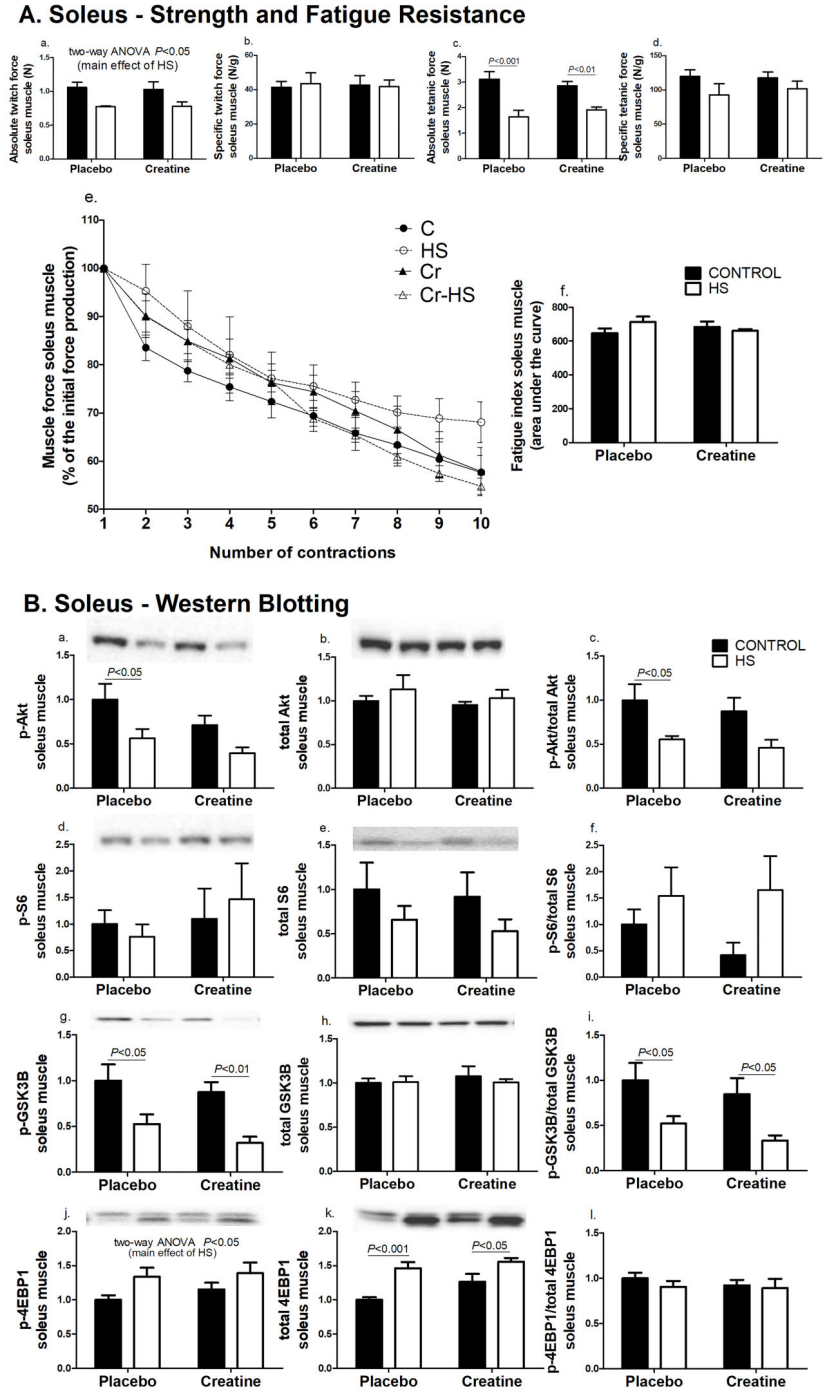
Strength, fatigue, and signaling pathways associated with protein synthesis in the soleus muscle. **A**, Parameters of strength and fatigue in the soleus muscle. The results were compared using two-way ANOVA and Bonferroni *post hoc* test. **Aa** is P<0.05 for C and Cr groups *vs* C-HS and Cr-HS groups (main effect of HS), using two-way ANOVA only (no statistical differences using the Bonferroni post-hoc test). **B**, Markers of signaling pathways associated to protein synthesis in the soleus muscle. Data are reported as means±SE on the basis of total protein loading as indicated by the Ponceau S measurements, n=6–8 animals. The results were compared using two-way ANOVA and Bonferroni *post hoc* test. **Bj** is P<0.05 for C and Cr groups *vs* C-HS and Cr-HS groups (main effect of HS), using two-way ANOVA only (no statistical differences using the Bonferroni *post hoc* test). C: control group; HS: hindlimb suspension group; Cr: creatine supplemented group; Cr-HS: creatine supplemented and hindlimb suspension group.

**Figure 2:**
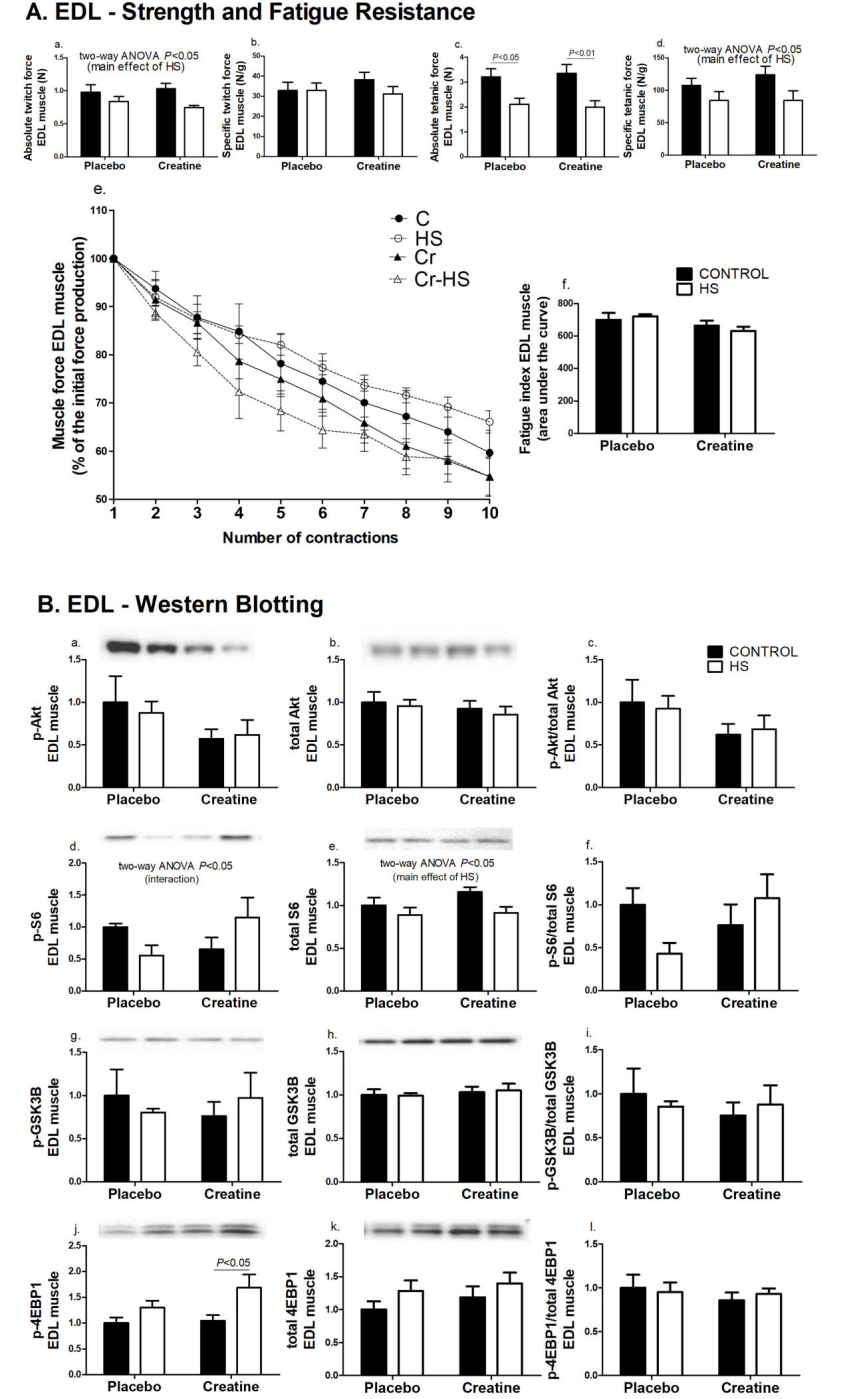
Strength, fatigue, and signaling pathways associated with protein synthesis in the extensor digitorum longus (EDL) muscle. **A**, Parameters of strength and fatigue in the EDL muscle. The results were compared using two-way ANOVA and Bonferroni *post hoc* test. **Aa** and **Ad** are P<0.05 for C and Cr groups *vs* C-HS and Cr-HS groups (main effect of HS), using two-way ANOVA only (no statistical differences using the Bonferroni *post hoc* test). **B**, Markers of signaling pathways associated with protein synthesis in the EDL muscle. Data are reported as means±SE on the basis of total protein loading as indicated by the Ponceau S measurements, n=6–8 animals. The results were compared using two-way ANOVA and Bonferroni *post hoc* test. **Bd** is P<0.05 for an interaction between the effects of HS and creatine supplementation, using two-way ANOVA only (no statistical differences using the Bonferroni *post hoc* test). **Be** is P<0.05 for C and Cr groups *vs* C-HS and Cr-HS groups (main effect of HS), using two-way ANOVA only (no statistical differences using the Bonferroni post-hoc test). C: control group; HS: hindlimb suspension group; Cr: creatine supplemented group; Cr-HS: creatine supplemented and hindlimb suspension group.

The absolute twitch force in the soleus and EDL muscles and the specific tetanic force in the EDL muscle were not changed as indicated by the inter-group analysis**.** However, HS decreased these parameters (P<0.05) compared with non-HS animals (Figure 1Aa and 2Aa). In the same way, the absolute tetanic forces in the soleus and EDL muscles were also significantly decreased by the hindlimb suspension (soleus muscle, Figure 1Ac: C *vs* HS, P<0.001 and Cr *vs* Cr-HS, P<0.01; EDL muscle, Figure 2Ac: C *vs* HS, P<0.05 and Cr *vs* Cr-HS, P<0.01). The specific twitch force and fatigue resistance did not change significantly in the soleus and EDL muscles due to either creatine supplementation or HS (Figure 1A and 2A).

### Protein synthesis- and degradation-associated signaling in soleus and EDL muscles

The content of p-Akt and the p-Akt/Akt total ratio were reduced by 45%, due to HS in the soleus muscle of the placebo group (C *vs* HS, P<0.05) and were not significantly changed by creatine supplementation (Figure 1Ba and 1Bc). S6 and p-S6 protein contents were not altered by HS or supplementation (Figure 1Bd,e,f). Decreases of p-GSK3B content (C *vs* HS, 47%, P<0.05 and Cr *vs* Cr-HS, 64%, P<0.01) and of p-GSK3B/GSK3B total ratio (C *vs* HS, 48% reduction, P<0.05 and Cr *vs* Cr-HS, 61% reduction, P<0.05) were reported in the placebo and creatine groups due to HS (Figure 1Bg and 1Bi, respectively). The p-4EBP1 content was not significantly changed in the inter-group analysis. However, two-way ANOVA indicated that HS significantly increased 4EBP1 phosphorylation (34% and 20% in the placebo and creatine groups, respectively, P<0.05) (Figure 1Bj). Total 4EBP1 content was also significantly increased in both groups (C *vs* HS, 46%, P<0.001, and Cr *vs* Cr-HS, 23%, P<0.05) (Figure 1Bk).

In the EDL muscle, the contents of phosphorylated and total Akt and GSK3B proteins did not change after the HS or creatine supplementation (Figure 2B). The content of p-S6 and the p-S6/S6 ratio were not significantly changed in the inter-group analysis. However, HS decreased S6 phosphorylation in the placebo group (by 44%) whereas creatine supplementation increased p-S6 content during HS (by 76%) (P<0.05) (Figure 2Bd). For total S6 content, two-way ANOVA indicated a decrease in both groups due to HS (11% in the placebo group and 21% in the creatine group, P<0.05) (Figure 2Be). The p-4EBP1 content was significantly increased (61%, P<0.05) in the HS group treated with creatine (Figure 2Bj).

### CSA of the soleus and EDL muscles fibers

**Figure 3:**
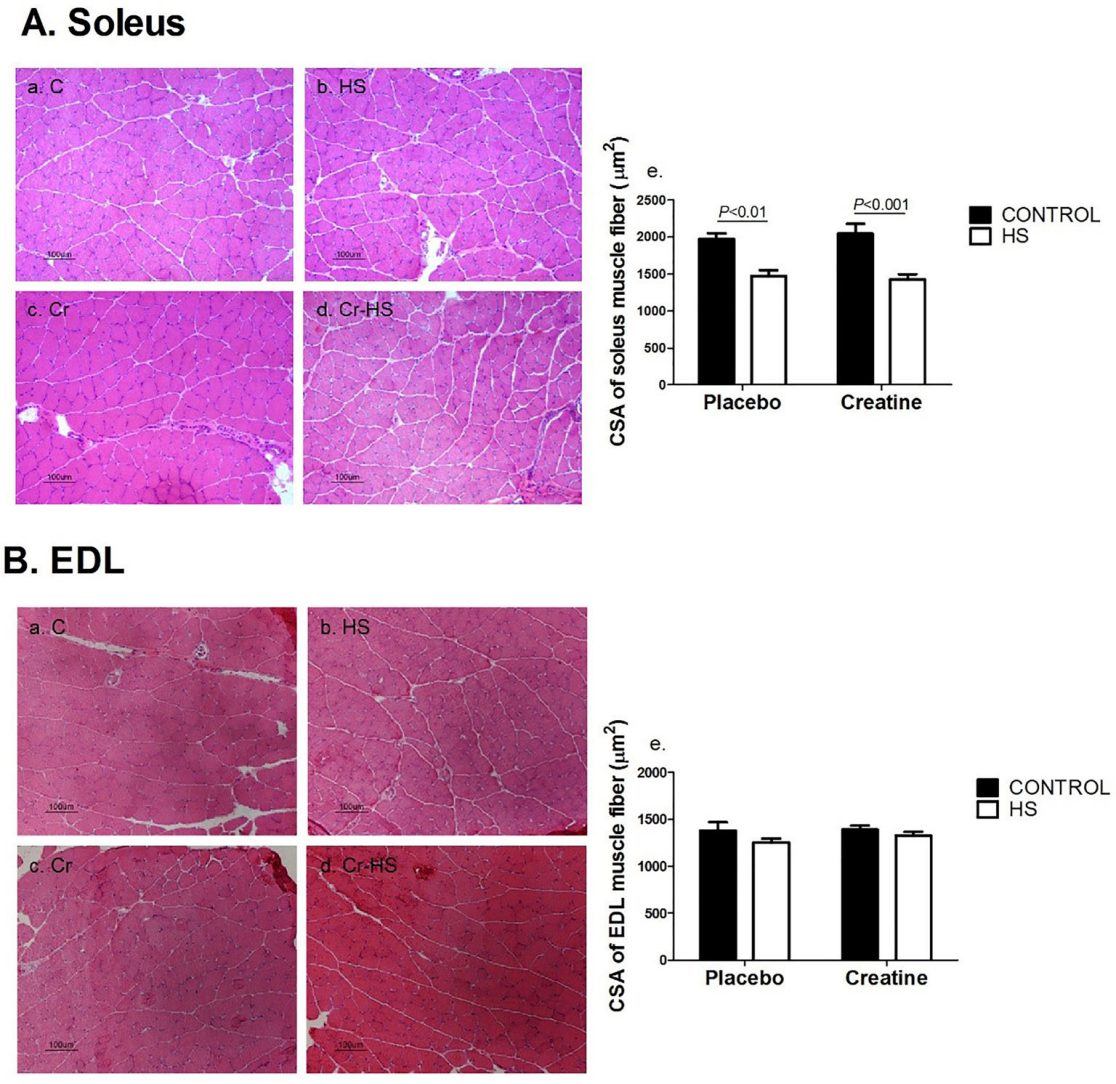
Cross-sectional areas (CSA) of the soleus and extensor digitorum longus (EDL) muscles fibers. **A**, Representative histological hematoxylin and eosin stained images of cross-sectional areas of the soleus muscle fibers and (**Ae**) CSA numerical data. Data are reported as means±SE, n=6–8 animals. The results were compared using two-way ANOVA and Bonferroni *post hoc* test. **B**, Representative histological hematoxylin and eosin stained images of cross-sectional areas of the EDL muscle fibers and (**Be**) CSA numerical data. Data are reported as means±SE, n=6-8 animals. The results were compared using two-way ANOVA and Bonferroni *post hoc* test. Magnification bar, 100 µm. C: control group; HS: hindlimb suspension group; Cr: creatine supplemented group; Cr-HS: creatine supplemented and hindlimb suspension group.

The CSA of the soleus muscle fibers was markedly decreased due to HS in both groups compared to non-HS animals (C *vs* HS, P<0.01 and Cr *vs* Cr-HS, P<0.001) by 25-30% (Figure 3A). The CSA of the EDL muscle fibers did not change due to HS in both groups compared to non-HS (Figure 3B). Creatine supplementation did not alter muscle fiber CSA in both soleus and EDL muscles.

### mRNA levels of *FST*, *MSTN*, *FAK*, *IGF*, *MGF*, *Akt*, *mTOR*, *atrogin-1*, and *MuRF1* in soleus and EDL muscles

**Figure 4:**
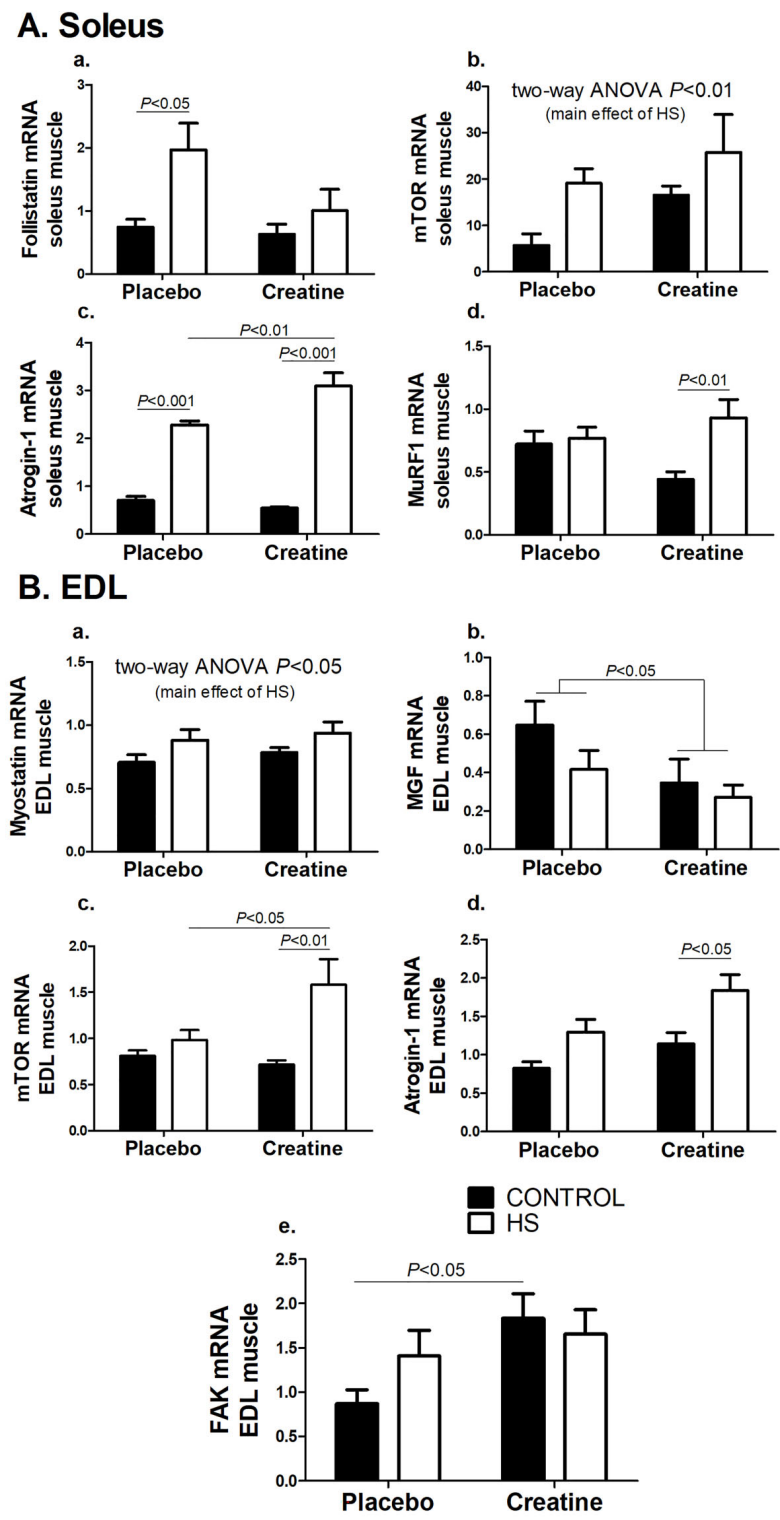
Expression of genes associated with protein synthesis and degradation signaling pathway in the soleus (**A**) muscle and in the EDL (**B**) muscle. Data are reported as means±SE, n=5–6 animals. The results were compared using two-way ANOVA and Bonferroni *post hoc* test. **Ab** and **Ba** are P<0.05 for C and Cr groups *vs* C-HS and Cr-HS groups (main effect of HS), using two-way ANOVA only (no statistical differences using the Bonferroni *post hoc* test). **Bb** is P<0.05 for C and HS groups *vs* Cr and Cr-HS groups (main effect of creatine), using two-way ANOVA only (no statistical differences using the Bonferroni *post hoc* test). C: control group; HS: hindlimb suspension group; Cr: creatine supplemented group; Cr-HS: creatine supplemented and hindlimb suspension group.

The expressions of *MSTN*, *FAK*, *IGF-1*, *MGF*, and *Akt* did not change in soleus muscle after the HS or with creatine supplementation (Supplementary Figure S1A). The expression of *FST* was increased due to HS in the C group (P<0.05) and creatine supplementation attenuated this change (Figure 4Aa). HS also resulted in an increased *mTOR* expression (P<0.01) (Figure 4Ab). The expression of *atrogin-1* was increased after the HS period in both groups (P<0.001) and it was further increased in the Cr-HS compared with the C-HS group (P<0.01) (Figure 4Ac). The expression of *MuRF1* was increased due to HS in the Cr group (Cr *vs* Cr-HS, P<0.01) (Figure 4Ad).

In the EDL muscle, the expressions of *FST*, *IGF*, *Akt*, and *MuRF1* also did not change after the HS period or creatine supplementation (Supplementary Figure S1B). However, HS increased the expression of *MSTN* in EDL muscle both in the placebo and creatine groups (P<0.05) (Figure 4Ba). Regarding *MGF* expression, a reducing effect of creatine supplementation was observed (P<0.05) (Figure 4Bb). *mTOR* expression was increased due to HS in the Cr group (P<0.01) (Figure 4Bc); the expression of this gene in the Cr-HS group was higher than in C-HS rats (P<0.05). The expression of *atrogin-1* increased after 5 days of HS in the Cr group (P<0.05) (Figure 4Bd). *FAK* expression increased in the creatine group compared with the placebo group (P<0.05) (Figure 4Be).

## Discussion

**Figure 5:**
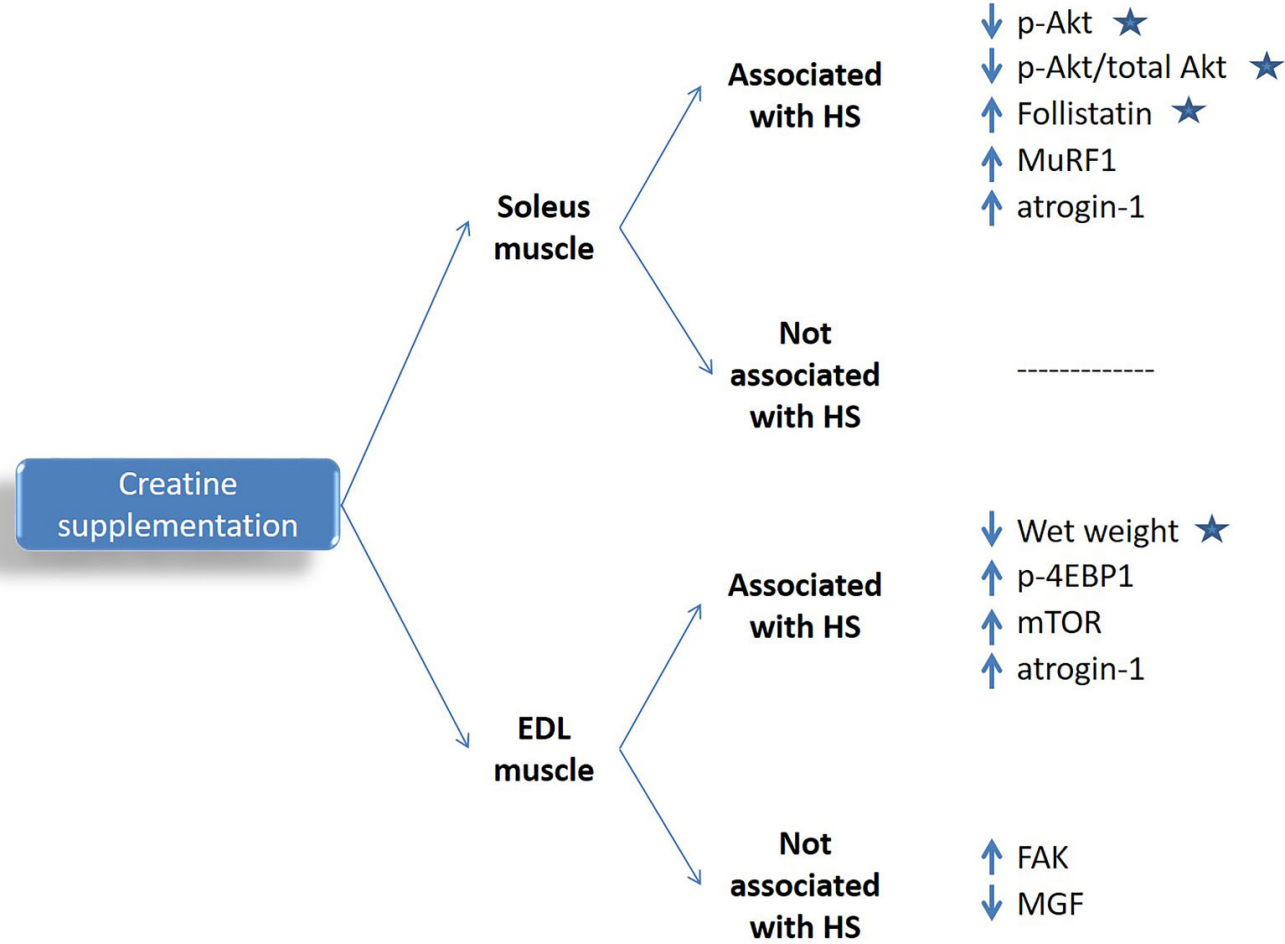
Main results observed in this study. Significant effects not associated with HS were observed in creatine supplemented animals only. EDL: extensor digitorum longus; HS: hindlimb suspension; p-Akt: phospo-protein kinase B content; MuRF1: muscle RING-Finger protein-1 expression; *atrogin*-1: muscle-specific F-box protein expression; p-4EBP1: eukaryotic translation initiation factor 4E (eIF4E) binding protein 1 content; *mTOR*: mammalian target of rapamycin expression; *FAK*: focal adhesion kinase expression; *MGF*: mechano growth factor expression; Stars indicate factors attenuated by creatine supplementation compared with the placebo group.

We investigated the effects of short-term creatine supplementation on skeletal muscle mass and strength and signaling pathways associated with protein synthesis or degradation in rats submitted to HS-induced atrophy. Despite the attenuating effects on protein metabolism signaling changes induced by HS, creatine supplementation, started concomitantly with HS, slighted prevented the decrease in skeletal muscle mass but had no effect on muscle CSA and strength after five days of muscle disuse (main results observed in this study are in Figure 5). The mentioned findings may be associated to the length of the supplementation period, the intensity of the muscle disuse atrophy, or the dose of creatine. Although, in the current study, creatine and phosphocreatine contents were not measured in the target muscles, the supplementation protocol used was reported to increase creatine content in type I fiber-rich muscles such as the soleus. The already described switch of type I to type II fibers during HS (31) may affect creatine accumulation in soleus muscle.

The content of total creatine is dependent on the skeletal muscle fiber type. Type II fibers have higher levels of creatine and phosphocreatine. Rodent type IIa and IIb fibers contain ∼32 mM phosphocreatine and 7 mM creatine whereas type I fibers have ∼16 mM phosphocreatine and 7 mM creatine. The EDL, a type II fiber-rich muscle, has a higher *K*_m_ (160 µM) and higher V_max_ (100 nmol·h^−1^·g^−1^ wet weight) compared with the type I fiber-rich soleus muscle (*K*_m_=73 µM and V_max_=77 nmol·h^−1^·g^−1^ wet weight), as calculated using ^14^C-labelled creatine. Therefore, creatine uptake and accumulation is a muscle fiber type-dependent process (9,32). Based on the above, two different muscles were studied: soleus (predominantly type I/oxidative/slow fibers) and EDL (predominantly type II/glycolytic/fast fibers). More intense atrophy due to HS was found in the soleus muscle based on the percentage of decrease in muscle mass and muscle fibers CSA, as also previously reported (1,2).

To evaluate protein synthesis signaling (Akt-mTOR-S6), we analyzed the phosphorylation and activation of the key upstream enzyme Akt and the phosphorylation of the S6 protein as a downstream signaling of mTOR activation. The activation of this pathway contributes to an increase in skeletal muscle mass by stimulating protein synthesis. This pathway also inhibits the activation of MuRF1 and *atrogin-1*, associated with the activation of the ubiquitin-proteasome degradation pathway, and promotes an inhibition of phosphorylation of GSK3b and 4EBP1 that can act as repressors of protein translation (33). In the soleus muscle, HS reduced p-Akt content, which is one of the main activators of mTOR complex. HS did not change p-S6 protein but enhanced p-4EBP1 protein. The lowered phosphorylation (and inhibition) of GSK3B followed the lower activation of its upstream kinase Akt. Activation of GSK3B leads to inhibition of eukaryotic translation initiation factor 2 beta (eIF2β) and to suppression of protein translation. Degradation pathway may be enhanced as indicated by increased *atrogin-1* mRNA expression. The lowered phosphorylation of Akt stops inhibiting FoXO protein and increases the *atrogin-1* content, which is an activating factor of the ubiquitin-proteasome pathway (34). Even though *FST* is associated with muscle hypertrophy (by inhibiting myostatin), our findings agreed with another study that reported an increase of this marker at the beginning of the HS period (35). Creatine supplementation reduced *FST* and increased *atrogin-1* expression during HS. Creatine supplementation had no marked hypertrophic effects in the soleus muscle. Conversely, it promoted an attenuation of the increase in *FST* expression and a greater increase in *MuRF1* expression due to HS.

Based on these findings, creatine would not be expected to markedly attenuate soleus muscle atrophy under the experimental conditions investigated. Indeed, soleus muscle atrophy, evaluated by muscle mass and fiber size, was not significantly different between groups. The mentioned findings also contributed to the lack of significant creatine effect on preventing leg muscle strength decrease induced by HS.

The EDL muscle suffered less atrophy than the soleus due to HS, which is in accordance with previous studies (1,2,27). This was reflected in the molecular analysis as well, with few changes induced by HS, including the reduction of p-S6 protein content and increase of *MSTN* expression. Myostatin is a member of the transforming growth factor-beta (TGF-β) family that acts on muscle mass control (36). This factor activates ubiquitin ligases and proteasome proteolysis (37), inhibiting protein synthesis through the Akt-mTOR-S6 pathway (38). In opposition to the soleus, promising effects of creatine on reduction of EDL muscle mass loss were found; the supplementation increased *FAK* expression. We speculate that the increase in the expression of FAK could produce an increase in the activation of this marker. Fak, an enzyme involved in the mechanical signaling associated with skeletal muscle hypertrophy, has the ability to phosphorylate p70S6K1 in Tyr 397, independently of Akt and mTOR (39). In the same line, creatine supplementation reversed the S6 phosphorylation trend changes, which tended to decrease with HS but it was increased by concomitant creatine supplementation. These effects were not enough to counteract EDL mass loss induced by HS in the period studied but they may represent an advantage for muscle recovery after facing an atrophy-promoting condition. An increased expression of *FAK* may not be important during HS because there is no mechanical loading to elicit marked *FAK* expression. However, after the unloading or disuse condition, the increased *FAK* expression promoted by creatine supplementation could be beneficial to enhance muscle recovery after mechanical loading is re-established (as a “reload priming”). In fact, Hespel et al. (22) concluded that creatine supplementation improves muscle mass recovery during rehabilitation using resistance training after a period of 2 weeks of immobilization in young healthy volunteers.

Along with our study, other authors investigated the effects of creatine supplementation starting concomitantly with distinct atrophic models such as dexamethasone treatment (17) or immobilization (19,22). Hespel et al. (22) reported that creatine does not attenuate mass loss and does not change protein synthesis and degradation markers (Akt, FoxO3a, and MuRF1) in dexamethasone-induced muscle atrophy (17). Crassous et al. (16) described similar outcomes after injury in rats muscle regeneration. The mentioned observations may be due to the long duration of the supplementation period, the pre-loading protocol, or the different muscle atrophy model. In our study, the short-term supplementation and the HS protocol used may not have provided enough time for the alterations in creatine content to exert a more noticeable effect. Aoki et al. (19) reported that creatine supplementation starting seven days before immobilization attenuates skeletal muscle wasting in rats.

Backx et al. (21) reported that, in healthy young males, creatine supplementation (20 g per day) for five days before and during seven days of leg immobilization does not preserve or attenuate the muscle mass loss or strength decrease. We reported similar observation in the HS animal model used that mimics the human bed rest condition. Safdar et al. (40) supplemented healthy young subjects with creatine for 10 days (without exercise or dietary intervention) and reported an increase in protein synthesis through activation of FAK and subsequent activation of Akt downstream pathways, following the increase in EDL muscle FAK activation described.

Some limitations of the study have to be mentioned such as the short period of supplementation, a single dose of creatine used, determination of muscle creatine content, severe muscle mass loss induced by HS, and lack of some mRNA expression and protein content measurements. These limitations, however, do not jeopardize the reported findings.

In conclusion, short-term creatine supplementation (5 g/kg b.w. per day for 5 days) changed protein metabolism signaling in soleus and EDL muscles. However, creatine supplementation only slightly attenuated the mass loss of both muscles and did not prevent the CSA and muscle strength decrease induced by HS for 5 days.

## Supplementary Material

Click here to view [pdf].

## References

[1] Bodine SC (2013). Disuse-induced muscle wasting. Int J Biochem Cell Biol.

[2] Marzuca-Nassr GN, Vitzel KF, De Sousa LG, Murata GM, Crisma AR, Rodrigues CF (2016). Effects of high EPA and high DHA fish oils on changes in signaling associated with protein metabolism induced by hindlimb suspension in rats. Physiol Rep.

[3] Boonyarom O, Kozuka N, Matsuyama K, Murakami S (2009). Effect of electrical stimulation to prevent muscle atrophy on morphologic and histologic properties of hindlimb suspended rat hindlimb muscles. Am J Phys Med Rehabil.

[4] Fujino H, Ishihara A, Murakami S, Yasuhara T, Kondo H, Mohri S (2009). Protective effects of exercise preconditioning on hindlimb unloading-induced atrophy of rat soleus muscle. Acta Physiol (Oxf).

[5] D'Antona G, Nabavi SM, Micheletti P, Di Lorenzo A, Aquilani R, Nisoli E (2014). Creatine, L-carnitine, and omega3 polyunsaturated fatty acid supplementation from healthy to diseased skeletal muscle. Biomed Res Int.

[6] Wall BT, van Loon LJ (2013). Nutritional strategies to attenuate muscle disuse atrophy. Nutr Rev.

[7] Devries MC, Phillips SM (2014). Creatine supplementation during resistance training in older adults-a meta-analysis. Med Sci Sports Exerc.

[8] Terjung RL, Clarkson P, Eichner ER, Greenhaff PL, Hespel PJ, Israel RG (2000). American College of Sports Medicine roundtable. The physiological and health effects of oral creatine supplementation. Med Sci Sports Exerc.

[9] Persky AM, Brazeau GA (2001). Clinical pharmacology of the dietary supplement creatine monohydrate. Pharmacol Rev.

[10] Wyss M, Kaddurah-Daouk R (2000). Creatine and creatinine metabolism. Physiol Rev.

[11] Bassit RA, Curi R, Costa Rosa LF (2008). Creatine supplementation reduces plasma levels of pro-inflammatory cytokines and PGE2 after a half-ironman competition. Amino Acids.

[12] Tarnopolsky MA (2011). Creatine as a therapeutic strategy for myopathies. Amino Acids.

[13] Guimarães-Ferreira L, Pinheiro CH, Gerlinger-Romero F, Vitzel KF, Nachbar RT, Curi R (2012). Short-term creatine supplementation decreases reactive oxygen species content with no changes in expression and activity of antioxidant enzymes in skeletal muscle. Eur J Appl Physiol.

[14] Menezes LG, Sobreira C, Neder L, Rodrigues-Junior AL, Martinez JA (2007). Creatine supplementation attenuates corticosteroid-induced muscle wasting and impairment of exercise performance in rats. J Appl Physiol (1985).

[15] Pearlman JP, Fielding RA (2006). Creatine monohydrate as a therapeutic aid in muscular dystrophy. Nutr Rev.

[16] Crassous B, Richard-Bulteau H, Deldicque L, Serrurier B, Pasdeloup M, Francaux M (2009). Lack of effects of creatine on the regeneration of soleus muscle after injury in rats. Med Sci Sports Exerc.

[17] Nicastro H, Gualano B, de Moraes WM, de Salles Painelli V, da Luz CR, dos Santos Costa A (2012). Effects of creatine supplementation on muscle wasting and glucose homeostasis in rats treated with dexamethasone. Amino Acids.

[18] Derave W, Van Den Bosch L, Lemmens G, Eijnde BO, Robberecht W, Hespel P (2003). Skeletal muscle properties in a transgenic mouse model for amyotrophic lateral sclerosis: effects of creatine treatment. Neurobiol Dis.

[19] Aoki MS, Lima WP, Miyabara EH, Gouveia CH, Moriscot AS (2004). Deleteriuos effects of immobilization upon rat skeletal muscle: role of creatine supplementation. Clin Nutr.

[20] Johnston AP, Burke DG, MacNeil LG, Candow DG (2009). Effect of creatine supplementation during cast-induced immobilization on the preservation of muscle mass, strength, and endurance. J Strength Cond Res.

[21] Backx EMP, Hangelbroek R, Snijders T, Verscheijden ML, Verdijk LB, de Groot LCPGM (2017). Creatine loading does not preserve muscle mass or strength during leg immobilization in healthy, young males: a randomized controlled trial. Sports Med.

[22] Hespel P, Op't Eijnde B, Van Leemputte M, Urso B, Greenhaff PL, Labarque V (2001). Oral creatine supplementation facilitates the rehabilitation of disuse atrophy and alters the expression of muscle myogenic factors in humans. J Physiol.

[23] Dirks ML, Backx EM, Wall BT, Verdijk LB, van Loon LJ (2016). May bed rest cause greater muscle loss than limb immobilization?. Acta Physiol (Oxf).

[24] Eijnde BO, Richter EA, Henquin JC, Kiens B, Hespel P (2001). Effect of creatine supplementation on creatine and glycogen content in rat skeletal muscle. Acta Physiol Scand.

[25] Casey A, Constantin-Teodosiu D, Howell S, Hultman E, Greenhaff PL (1996). Creatine ingestion favorably affects performance and muscle metabolism during maximal exercise in humans. Am J Physiol.

[26] Greenhaff PL, Bodin K, Soderlund K, Hultman E (1994). Effect of oral creatine supplementation on skeletal muscle phosphocreatine resynthesis. Am J Physiol.

[27] Marzuca-Nassr GN, Murata GM, Martins AR, Vitzel KF, Crisma AR, Torres RP (2017). Balanced diet-fed Fat-1 transgenic mice exhibit lower hindlimb suspension-induced soleus muscle atrophy. Nutrients.

[28] Fortes MA, Pinheiro CH, Guimaraes-Ferreira L, Vitzel KF, Vasconcelos DA, Curi R (2015). Overload-induced skeletal muscle hypertrophy is not impaired in STZ-diabetic rats. Physiol Rep.

[29] Bodine SC, Baar K (2012). Analysis of skeletal muscle hypertrophy in models of increased loading. Methods Mol Biol.

[30] Fortes MA, Marzuca-Nassr GN, Vitzel KF, da Justa Pinheiro CH, Newsholme P, Curi R (2016). Housekeeping proteins: How useful are they in skeletal muscle diabetes studies and muscle hypertrophy models?. Anal Biochem.

[31] Baldwin KM, Haddad F, Pandorf CE, Roy RR, Edgerton VR (2013). Alterations in muscle mass and contractile phenotype in response to unloading models: role of transcriptional/pretranslational mechanisms. Front Physiol.

[32] Willott CA, Young ME, Leighton B, Kemp GJ, Boehm EA, Radda GK (1999). Creatine uptake in isolated soleus muscle: kinetics and dependence on sodium, but not on insulin. Acta Physiol Scand.

[33] Bonaldo P, Sandri M (2013). Cellular and molecular mechanisms of muscle atrophy. Dis Model Mech.

[34] Bodine SC, Baehr LM (2014). Skeletal muscle atrophy and the E3 ubiquitin ligases MuRF1 and MAFbx/atrogin-1. Am J Physiol Endocrinol Metab.

[35] Stevenson EJ, Giresi PG, Koncarevic A, Kandarian SC (2003). Global analysis of gene expression patterns during disuse atrophy in rat skeletal muscle. J Physiol.

[36] McPherron AC, Lawler AM, Lee SJ (1997). Regulation of skeletal muscle mass in mice by a new TGF-beta superfamily member. Nature.

[37] Lokireddy S, McFarlane C, Ge X, Zhang H, Sze SK, Sharma M (2011). Myostatin induces degradation of sarcomeric proteins through a Smad3 signaling mechanism during skeletal muscle wasting. Mol Endocrinol.

[38] Trendelenburg AU, Meyer A, Rohner D, Boyle J, Hatakeyama S, Glass DJ (2009). Myostatin reduces Akt/TORC1/p70S6K signaling, inhibiting myoblast differentiation and myotube size. Am J Physiol Cell Physiol.

[39] Klossner S, Durieux AC, Freyssenet D, Flueck M (2009). Mechano-transduction to muscle protein synthesis is modulated by FAK. Eur J Appl Physiol.

[40] Safdar A, Yardley NJ, Snow R, Melov S, Tarnopolsky MA (2008). Global and targeted gene expression and protein content in skeletal muscle of young men following short-term creatine monohydrate supplementation. Physiol Genomics.

